# Detection of human papillomaviruses in paired healthy skin and actinic keratosis by next generation sequencing

**DOI:** 10.1016/j.pvr.2020.100196

**Published:** 2020-03-25

**Authors:** Luisa Galati, Rosario Nicola Brancaccio, Alexis Robitaille, Cyrille Cuenin, Fabiola Luzi, Gianna Fiorucci, Maria Vincenza Chiantore, Nadia Marascio, Giovanni Matera, Maria Carla Liberto, Maria Gabriella Donà, Paola Di Bonito, Tarik Gheit, Massimo Tommasino

**Affiliations:** a International Agency for Research on Cancer-World Health Organization, Lyon, France; b Plastic and Reconstructive Surgery, San Gallicano Dermatologic Institute IRCCS, Rome, Italy; c Department of Infectious Diseases, EVOR Unit, Istituto Superiore di Sanità, Rome, Italy; d Institute of Molecular Biology and Pathology, Consiglio Nazionale delle Ricerche, Rome, Italy; e "Magna Graecia” University, Catanzaro, Italy; f STI/HIV Unit, San Gallicano Dermatologic Institute IRCCS, Rome, Italy

**Keywords:** HPV, NGS, Actinic keratosis, Cutaneous squamous cell carcinoma (cSCC), Epidemiology, HPV, Human papillomavirus, AK, Actinic keratosis, HS, healthy skin, cSCC, cutaneous squamous cell carcinoma, EV, *epidermodysplasia verruciformis*, RAxML, Randomized Axelerated Maximum Likelihood, EPA, Evolutionary placement algorithm

## Abstract

Actinic keratosis (AK) arises on photo-damaged skin and is considered to be the precursor lesion of cutaneous squamous cell carcinoma (cSCC). Many findings support the involvement of β human papillomaviruses (HPVs) in cSCC, while very little is known on γ HPV types. The objective of this study was to characterize the spectrum of PV types in healthy skin (HS) and AK samples of the same immunocompetent individuals using next generation sequencing (NGS). Viral DNA of 244 AK and 242 HS specimens were amplified by PCR using two different sets of primers (FAP59/64 and FAPM1). Purified amplicons were pooled and sequenced using NGS. The study resulted in the identification of a large number of known β and γ PV types. In addition, 27 putative novel β and 16 γ and 4 unclassified PVs were isolated. HPV types of species γ-1 (e.g. HPV4) appeared to be strongly enriched in AK versus HS. The NGS analysis revealed that a large spectrum of known and novel PVs is present in HS and AK. The evidence that species γ-1 HPV types appears to be enriched in AK in comparison to HS warrants further studies to evaluate their role in development of skin (pre)cancerous lesions.

## 1 Introduction

Cutaneous squamous cell carcinoma (cSCC) arises from progression of the precursor lesion, actinic keratosis (AK), which develops on photo-damaged skin [1]. Ultraviolet (UV) radiation exposure is the main risk factor in the development of AK and cSCC [2,3]. Skin lesion development is also positively associated with fair skin, advanced age and immunosuppression [4]. The concept that impairment of the immune system favors cSCC development supports the involvement of an infectious agent, such as the epitheliotropic human papillomaviruses (HPVs). HPVs are circular double-stranded DNA viruses infecting mucosal and cutaneous epithelia. To date, more than 200 HPV genotypes have been fully characterized and classified into five genera (α, β, γ, mu and nu papillomaviruses) according to the nucleotide sequences of the ORF encoding for the major capsid protein L1 [5] (https://pave.niaid.nih.gov/). A subgroup of α-genus HPV types, referred to as mucosal high-risk (HR) HPV types, has been clearly associated with human carcinogenesis [6,7]. Twelve HR HPV types, namely 16, 18, 31, 33, 35, 39, 45, 51, 52, 56, 58, 59 have been classified as Group 1, carcinogens to humans, by the International Agency for Research on Cancer (IARC) [8]. In addition to the mucosal HR HPV types, epidemiological and biological studies support the role of β-genus HPV types in the development of cSCC, together with UV radiation [9]. The first β HPV types, HPV5 and 8, were identified in skin lesions of *epidermodysplasia verruciformis* (EV) patients, who are highly susceptible to β HPV infection and UV-induced cSCC [10,11]. Accordingly, IARC has classified β HPV 5 and HPV 8 as “possibly carcinogenic” agents (Group 2B) in EV patients [8]. Since their isolation, additional 52 β HPV types have been characterized so far, which are subdivided into 5 species, β1-5 (http://www.nordicehealth.se/hpvcenter/reference_clones/) [12] and are abundantly present on the skin of healthy individuals [[13], [14], [15], [16]]. In addition to EV patients, β HPV types appear to be involved in cSCC development also in immunocompromised individuals, such as organ transplant recipients (OTR), as well as in elderly general population [[17], [18], [19], [20]]. In contrast to α HR HPV types, the presence of β HPVs does not appear to be required for the maintenance of the malignant phenotype [21]. Studies in *in vivo* experimental models provide evidence for a “hit-and-run” mechanism of β HPVs involvement in UV-induced skin carcinogenesis [1,[21], [22], [23]]. Accordingly β HPV prevalence and viral load decrease during carcinogenesis process in humans, being significantly higher in AK than in cSCC [24,25]. Other cutaneous HPV types that are frequently detected in skin are the ones that belong to γ genus. They represent the largest clade within the *Papillomaviridae* family. Almost 100 γ HPV types subdivided into 27 species have been fully characterized so far. No clear association of γ HPVs with malignant lesions has been demonstrated, although biological studies showed that E6 and E7 proteins from some γ HPVs display *in vitro* transforming activities [26].

To gain new insights on the presence of a broad spectrum of β and γ HPV types in healthy skin (HS) and actinic keratosis (AK) of the same individual, we used different PCR protocols [[27], [28], [29]] combined with Next Generation Sequencing (NGS). We used the well-validated broad-spectrum FAP primers and an updated version FAPM1 primers. The latter set of primers was designed taking into consideration DNA sequences of recently characterized beta HPV types [[27], [28], [29]]. The results revealed the presence of a large spectrum of β and γ HPV types. Interestingly, species γ-1 HPV types appear to be more represented in AK than in HS.

## 2 Materials and methods

### 2.1 Patient selection, sample collection and DNA extraction

Skin scraping samples (HS and AK) from a previous study aimed to determine the prevalence of cutaneous HPVs in AK lesions by using a sensitive Luminex based-beads multiplex assay were used in the present analysis [30]. Skin samples were collected from 244 immunocompetent patients (142 men and 102 women in age range 48–94 years) with a diagnosis of AK attending the dermatology outpatient clinic of the National Institute for Health, Migration and Poverty (NIHMP) in Rome (Italy). A total of 488 individual samples were collected by scraping the lesions and, separately, the healthy skin of the glabellar region with a sterile spatula. The majority of the AK lesions were in the head region (n = 221) while others were located in the limbs (n = 5) and other anatomical sites (n = 18). In the present analysis, two HS samples were excluded due to the shortage of the residual sample. Samples were stored at −80 °C until treatment with proteinase K for 4 h at 50 °C in 10 mM Tris-HCl pH 8.0, 50 mM NaCl, 5 mM EDTA, 1 mM DTT, 0.5% SDS (0.4 ml/sample). Nucleic acids, extracted by magnetic silica using the automated system NucliSENS EasyMag (Biomérieux, France) according to the manufacturer's directions, were analyzed at IARC (Lyon, France) by NGS. Written informed consent was obtained from all enrolled patients. The study was approved by the Ethical Commettes of both NIHMP (2014) and San Gallicano Dermatologic Institute (CE943/17).

### 2.2 PCR amplification and amplicon purification

Extracted DNA was amplified using two different sets of primers; the consensus primer pair FAP (FAP59\FAP64) targeting the 5′end of the L1 ORF as previously reported [27], and a new set of degenerated FAP primers (FAPM1 primer mix) as previously described by Brancaccio et al. [29]. Both FAP and FAPM1 primers target a region of the L1 ORF yielding an amplicon of about 480 bp. PCR amplicons were visualized by electrophoresis on a 2% agarose gel and purified using QIAquick gel extraction purification kit according to the manufacturer's instructions (QIAGEN, Hilden, Germany).

#### 2.2.1 Library preparation and NGS

Purified PCR amplicons were divided into twelve different pools as described in Table 1. Each pool was obtained using 2 μl of each purified PCR product. Before library preparation, one additional purification step was performed in each pool to remove any residual contaminants using the Agencourt AMPure XP PCR purification kit with a beads ratio of 1.8 X (Beckman Coulter) according to the manufacturer's instructions.

**Table 1:** **Description of the NGS pools.** All the PCR products (n = 685) were grouped in 12 NGS pools according to the type of skin sample and PCR protocol applied as indicated in the table. For pools 1–3, 2–4, 5–7, 6 both HS and AK specimens of the same individuals gave a PCR product with indicated primers. In contrast the remaining pools include PCR products of unpaired AK (9−10) or HS (11–12), since the matched skin samples were negative with indicated PCR protocols.

NGS pool	PCR protocol	Specimen (AK or HS)∗	Total number
1	FAP59/64	AK	71
3	FAP59/64	HS	71
2	FAP59/64	AK	70
4	FAP59/64	HS	70
5	FAPM1	AK	53
7	FAPM1	HS	53
6	FAPM1	AK	53
8	FAPM1	HS	53
9	FAP59/64	AK	34
10	FAPM1	AK	16
11	FAP59/64	HS	41
12	FAPM1	HS	100

∗ AK, actinic keratosis; HS, healthy skin.

Twelve libraries were prepared using the Nextera ™ DNA Flex Library preparation kit (Illumina, San Diego, CA, US). Illumina MiSeq dual-indexed adapters (Illumina, San Diego, CA, US) were added to each of the PCR pools. The library sizes were checked using the Bioanalyzer 2100 Expert (Agilent) using high sensitivity DNA assay. NGS analysis was performed on 4 nM of DNA pooled library using an Illumina MiSeq instrument (2 × 150 paired-end reads with the Illumina MiSeq kit v3). In order to enrich the diversity of the libraries, 10% of PhiX (Illumina, San Diego, CA, US) was added to the NGS reaction.

#### 2.2.2 Bioinformatic analysis of NGS sequences

The bioinformatic workflow includes common data preprocessing steps for quality control and filtering. Then, data complexity is reduced before the identification of the PV-related sequences. Groups of sequences are defined based on similarity between identified sequences and available PVs sequences in the NCBI database. De-novo assembly is then performed to reconstruct the full amplified region covered by several primers systems. Finally, the reconstructed sequences are taxonomically classified based on two independent methodologies: alignment-based, and homology-based, respectively, before generation of diverse output reports. Details of the bioinformatic pipeline named “PVAmpliconFinder” and parameters used can be found in (https://github.com/IARCbioinfo/PVAmpliconFinder).

All the results in this study are based on the identification of the sequences following the homology-based classification using the evolutionary placement algorithm (EPA) in RAxML (Randomized Axelerated Maximum Likelihood) [31,32] (henceforth referred to as RAxML-EPA). Only the longest sequence was considered for RAxML-EPA classification when several singlets or contigs were available.

## 3 Results

### 3.1 HPV DNA PCR amplification and NGS analyses

Amplicons of the expected size were detected in 75.2% (182/242) and in 85.1% (206/242) of HS samples, using FAP59/FAP64 and FAPM1 protocols, respectively. A PCR product of the expected size was detected in 71.2% (175/244) and in 50% (122/244) of the AK samples by using the primer sets FAP59/FAP64 and the novel FAPM1, respectively.

PCR amplicons generated by the use of the two different sets of primers on HS and AK DNA samples were pooled as shown in method section and sequenced using the NGS platform MiSeq Illumina. The NGS analysis generated a total of 1,209,249 reads. A total of 1,208,356 of the reads was considered for further analysis after quality trimming, and chimeric PCR sequence removal. All of them, were identified as related to PVs sequences (>99% of reads). Each read was matched against the National Center for Biotechnology Information (NCBI) sequences database by means of BLAST algorithm and assigned to its closest PV types.

The different PV sequences were analyzed following the official taxonomic HPV classification based on the similarity in L1 ORF [5].

**Fig. 1:**
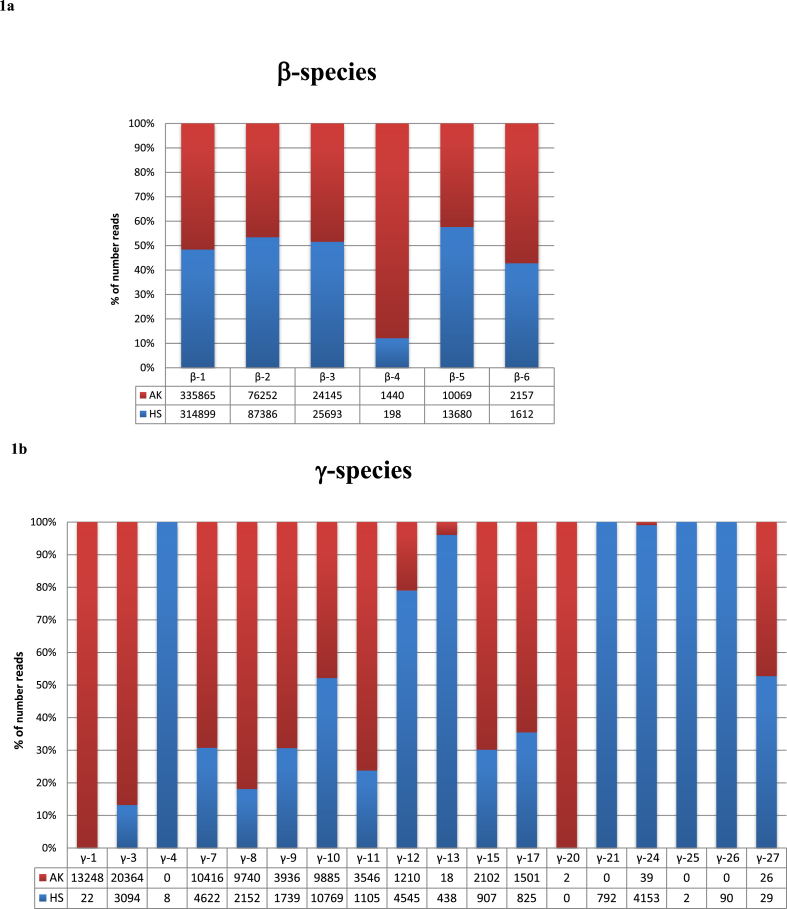
**Detected PV species in HS and AK samples.** Relative abundance of species (%) beta (1a) and gamma (1b) according to RaxML-EPA classification in both healthy skin (HS) and actinic keratosis (AK) samples are shown.

Data analysis obtained using RAxML-EPA, a method that offers an accurate classification of short PV fragments, reported that the 1,208,356 reads analyzed comprised 1,204,447 (99.7%) reads from known PVs (≥90% of identity with L1 ORF of any known PV), while the remaining reads (n = 3909) corresponded to novel putative PVs (<90% of identity with L1 ORF of any known PV). The majority of the reads (81.1%, 976,693 reads) corresponded to β PVs, followed by γ (17.3%, 208,932 reads) and α types (0.01%, 121 reads) (Table 2). According to RaxML-EPA analysis of known PV sequences, the major number of reads were related to human PVs (n = 1,181,306), while the remaining were closely related to non-human PVs (total non-human reads: 23,141) i.e. *Macaca fascicularis PV type 2* (MfPV2) belonging to β-6 genus (3769 reads), *Macaca mulatta papillomavirus type 5* (MmPV5) (671 reads) that is classified into the γ genus, and *Erethizon dorsatum papillomavirus 2* (EdPV2) (18,701 reads), a new PV still unclassified (Table S1). In summary, 1,204,447 reads are representative of 1786 PVs sequences. As a specific PV sequence can be represented more than one time among the different pools, or different PV sequences can be assigned to the same PV type, thus 1786 PV sequences corresponded to 195 distinct PV types (Table 2 and Table S1). Of the 195 PV types, 93 resulted to be officially recognized, namely 2 sequences from α-2 species, 49 sequences from β 1–6 and 42 sequences spreading into 18 γ species (Fig. 1). The remaining sequences corresponded to 12 unclassified-β and 89 unclassified-γ PVs. Only one sequence remained unclassified and was assigned by RAxML-EPA analysis to a divergent and unclassified EdPV2 sequence (Tables 2 and S1).

**Table 2:** **Known and putative novel PVs sequences in healthy skin (HS) and actinic keratosis (AK) samples**. The number of sequences and corresponding reads are reported for alpha, beta, gamma and unclassified PVs, stratified according to the primer set, by RAxML-EPA taxonomic classification.

PV genus	Known PVs sequences N (reads)	KNOWN PVs				Putative new PV sequences N (reads)	UNKNOWN PVs			
		HS		AK			HS		AK	
		FAP59/64 PV sequences N (reads)	FAPM1 PV sequences N (reads)	FAP59/64 PV sequences N (reads)	FAPM1 PV sequences N (reads)		FAP59/64 Unique PV sequences (N reads)	FAPM1 Unique PV sequences (N reads	FAP68/64 Unique PV sequences (N reads)	FAPM1 Unique PV sequences (N reads)
**alpha**	**2 (121)**	0 (0)	2 (54)	0 (0)	1 (67)	**0**	0 (0)	0 (0)	0 (0)	0 (0)
**beta**	**61 (976,693)**	54 (311,187)	60 (174,731)	54 (253,407)	57 (237,368)	**27 (3459)**	9 (1878)	6 (206)	6 (675)	6 (700)
**gamma**	**131 (208,932)**	67 (46,262)	91 (39,306)	85 (61,128)	87 (62,236)	**16 (376)**	4 (153)	7 (117)	0 (0)	5 (106)
**unclassified PV**	**1 (18,701)**	1 (1510)	1 (8678)	1 (2568)	1 (5945)	**4 (74)**	0 (0)	0 (0)	1 (14)	3 (60)
**Total**	**195 (1,204,447)**	122 (358,959)	154 (222,769)	140 (317,103)	146 (305, 616)	**47 (3909)**	13 (2031)	13 (323)	7 (689)	14 (866)

#### 3.1.1 Known PV sequences in HS and AK

We next compared the distribution of the different PVs sequences in AK and HS. The distribution of all known HPV types detected in HS and AK is shown in Table S1 and the relative abundance is in Fig. 1. Regarding α HPV types, the small number of reads (n = 121) generated exclusively by the FAPM1 protocol corresponded to sequences of the two closely related cutaneous HPVs 3 and 28. However, most of the reads were from HPV28, which was equally distributed in HS and AK (Table S1). Reads of β HPV sequences were approximately equally represented in HS and AK (485,918 and 490,775 respectively), with the exception of β-4 species, represented by HPV type 92 only. For this the number of reads was more abundant in AK than HS (1440 vs 198 reads) (Fig. 1a, Table S1).

Regarding the γ HPV types, reads for the different species were differently detected in HS (85,568 reads) and AK (123,364 reads) samples. The relative abundance of γ-types was in some cases higher in AK than the relative abundance in HS (i.e. γ-1, γ-3, γ-7, γ-8, γ-9, γ-11, γ-15,γ-17) and vice versa in other cases (i.e. γ-12, γ-13, γ-21, γ-24) (Fig. 1b). Moreover, for the majority of the species only a small number of reads were detected in HS and AK (i.e γ −4, γ-20 and γ −25). Interestingly, for γ-1 species 600 fold difference in number of reads was observed in AK versus HS (13,248 and 22 reads, respectively) (Fig. 1b and Table S1). The majority of these γ-1 reads corresponded to HPV4 (13,207 reads) (Table S1).

#### 3.1.2 Putative novel PVs

Finally, 3909 (0.3%) reads generated a total of 47 putative novel PV types, since the fragment sequence showed less than 90% similarity to L1 ORF of any known PVs. As per the RaxML-EPA classification, the majority of reads were closely related to human PVs (3827 reads). Of the 3827 reads, a substantial number of reads were closely related to β-HPVs (3457 reads), and γ-HPVs (370 reads). Whereas, for the non-human PVs, out of 82 reads, 74 reads were from the unclassified PVs category (Table S2).

Among the unknown PV sequences, 26 (55.3%) putative novel sequences were found in HS and 21 (44.7%) in AK specimens, respectively (Tables 2 and S2). The FAPM1 primers detected a slightly higher number of putative novel HPV sequences than FAP59/64, i.e. 27 and 20, respectively (Table 2).

Using RAxML-EPA classification, 15 putative novel β PVs and 11 putative novel γ PVs were isolated from HS samples, whereas 12 novel β PVs and 5 γ PVs were isolated from AK samples. The remaining 4 putative novel PVs, isolated from AK samples, remained unclassified (Table 2).

The FAPM1 protocol allowed the isolation of a total of 12 novel γ PVs in AK and HS samples, while the FAP protocol allowed the isolation of 4 γ PVs only in HS samples (Tables 2 and S2).

Putative new PV types in AK samples were related to HPV5, 21 (belonging to species β-1), HPV15, 22, 23, 120 (species β-2) and HPV130 (species γ-10) (Table S2). In HS samples, the new PV sequences were related to species β-1 (HPV5, 21, 24), β-2 (HPV22, 23, 38), γ-10 (HPV133) and γ-27 (HPV201).

## 4 Discussion

Cutaneous HPV types spread over all five HPV genera and are abundantly present in normal skin. Since several lines of evidence support the role of β HPV types in favoring the UV-induced skin carcinogenesis, epidemiological studies focused mostly only on β-HPV detection in pre-malignant and malignant skin lesions. In contrast to β HPV types, the biology and epidemiology of γ HPV types have been poorly investigated so far. In a recent study, we have determined the prevalence of 46 β and 52 γ HPV types in HS and AK of the same individuals who have been included in this study [30]. Dona et al. reported that the prevalence of most of the β and γ HPV types decreased from HS to AK, suggesting that cutaneous HPVs may play a role at early phase of AK lesion development and can be lost once the lesion is fully established [30]. To have a more accurate scenario on the HPV types present in HS and AK, we have re-analyzed the same cohort performing a broad spectrum analysis of cutaneous HPV types by NGS. Our data confirmed previous findings that β1 and β2 are the most represented species in both HS and AK, followed by β3, β4 and β5 [30,33]. It is not yet clear why the β4 and β5 HPV types are poorly present in the skin. One possible hypothesis is that these HPVs have a low efficiency in persisting in the host skin. Alternatively, β3-5 HPV types may preferentially infect other anatomical sites than the skin. In support of this hypothesis, it has been shown that β3 are more prevalent in mucosal epithelia than in the skin [33,34]. In agreement with the epidemiological data, functional studies revealed that β3 HPV types 49 shares some biological properties with the mucosal HR HPV16 *in vitro* and *in vivo* experimental models [[35], [36], [37]].

The γ genus is the largest clade within the Papillomaviridae family and the improvement of sequencing methods has led to the identification of many novel γ types over the last years [[38], [39], [40]]. The γ PVs can be found in common warts, in skin tumors and AK samples, as well as in normal skin [30,[41], [42], [43]]. Our NGS-based analysis revealed that almost all γ species were represented in HS and AK, except for γ-2, γ-5, γ-6, γ-14, γ-18 and γ-23. In addition to this, a relevant number of γ species that are not yet classified by the HPV reference center was also found. However, one limitation of our study is that it does not provide information on the distribution of specific HPV types in HS and AK in single individuals, since PCR amplicon pools were generated for the next-generation sequencing.

Interestingly, although most of the β and γ HPV types were equally represented in HS and AK samples, γ-1 HPV4 was strongly enriched in AK samples versus HS. Similar results were observed in our recent study where HPV detection was performed by a highly specific genotyping assay [30]. In this study, using the same samples, the number of reads that correspond to HPV4 was indeed higher in AK (13,207 reads) in comparison to HS (20 reads). These findings suggest a possible link between HPV4 infection and AK development. Alternatively, this specific γ HPV type might have some biological differences with respect to the other γ HPV types, for instance it could benefit from the tissue alterations occurring in AK for completion of its life cycle. Additional work is required to further evaluate these two hypothesis. So far, it has been reported that HPV4 is associated with the development of mosaic warts [44,45]. Regarding HPV4 biological properties, it has been shown that its E7 is able to degrade pRb [46], as the mucosal HR HPV E7s. Mutations in the EVER1 or EVER2 have been associated with high susceptibility to cutaneous HPV infection and the development of the EV disease [47]. It has been recently shown that EVER1/EVER2 form a complex with the encoding the pleiotropic factor calcium- and integrin-binding protein 1 (CIB1) [48]. Interestingly, HPV4 E8 interacts with CIB1, suggesting that this virus may interfere with EVER1/EVER2/CIB1-dependent restriction of viral infection.

In the present study we identify 195 known HPV types and in addition to this, using different PCR protocols combining with NGS, we identified 47 putative novel PVs. The analysis of these putative novel PVs revealed that they are related to 27 β, 16 γ and 4 unclassified PVs. Of which, 1 β PV, 1 γ and 4 unclassified PVs were non-human PVs. The classification of these PVs as animal types relies on short DNA sequences. The complete characterization of the entire genome could result in a different classification as novel HPVs. However, the possibility that these PVs represent a contamination from domestic animals or have crossed the species should be also taken into consideration.

Interestingly, our study led to the identification of 15 putative novel β-2 HPV types. β-2 species includes HPV38, which displays *in vitro* and *in vivo* transforming properties. Also HPV38 has been found significantly associated with the risk of cSCC in a recent meta-analysis [49].

## 5 Conclusions

In summary, using a robust strategy based on the use of degenerated primers and NGS technology this study expanded our knowledge and efficiently depicted the PV population in AK and HS sample. Moreover, it allowed the detection of putative novel PVs, although the identification of novel PV types or species can only be definitively confirmed by sequencing the whole L1 ORF. Finally, it showed that some γ HPV types (e.g., HPV4) are enriched in AK vs. HS, and might thus play a role in skin carcinogenesis, thus deserving further *in vivo* and *in vitro* investigations.

## Funding

The study was supported by Fondation ARC pour la recherche sur le cancer, France (no. PJA 20151203192) (https://www.fondation-arc.org/espace-chercheur) and the Institut National de la Santé et de la Recherche Médicale (no. ENV201610) France (https://www.eva2.inserm.fr/EVA/jsp/AppelsOffres/CANCER/) to MT.

## CRediT authorship contribution statement

**Luisa Galati:** Writing - original draft. **Rosario Nicola Brancaccio:** Methodology. **Alexis Robitaille:** Formal analysis. **Cyrille Cuenin:** Methodology. **Fabiola Luzi:** Conceptualization. **Gianna Fiorucci:** Conceptualization. **Maria Vincenza Chiantore:** Conceptualization. **Nadia Marascio:** Formal analysis. **Giovanni Matera:** Formal analysis. **Maria Carla Liberto:** Formal analysis. **Maria Gabriella Donà:** Conceptualization. **Paola Di Bonito:** Writing - original draft, Conceptualization. **Tarik Gheit:** Writing - original draft. **Massimo Tommasino:** Writing - original draft.

## Declaration of competing interest

The authors declare that they have no known competing financial interests or personal relationships that could have appeared to influence the work reported in this paper.

## Appendix A Supplementary data

Table S1 Known PV types in healthy skin (HS) and actinic keratosis (AK) samples according to RAxML-EPA taxonomic classification. PV types (n = 195) and corresponding NGS reads are reported for PV species stratified according to the PCR primer sets and skin specimen. Table S1

Table S2 Putative new PVs in healthy skin (HS) and actinic keratosis (AK) samples according to RAxML-EPA taxonomic classification. The 47 putative new PVs and corresponding NGS reads are listed according to the PCR primer sets and skin specimens. Table S2
